# Handheld multispectral imager for quantitative skin assessment in low-resource settings

**DOI:** 10.1117/1.JBO.25.8.082702

**Published:** 2020-08-04

**Authors:** Luigi Belcastro, Hanna Jonasson, Tomas Strömberg, Rolf B. Saager

**Affiliations:** Linköping University, Department of Biomedical Engineering, Linköping, Sweden

**Keywords:** multispectral imaging, spatial frequency domain imaging, low-resource settings, digital micromirror device, skin, phantoms

## Abstract

**Significance:** Spatial frequency domain imaging (SFDI) is a quantitative imaging method to measure absorption and scattering of tissue, from which several chromophore concentrations (e.g., oxy-/deoxy-/meth-hemoglobin, melanin, and carotenoids) can be calculated. Employing a method to extract additional spectral bands from RGB components (that we named cross-channels), we designed a handheld SFDI device to account for these pigments, using low-cost, consumer-grade components for its implementation and characterization.

**Aim:** With only three broad spectral bands (red, green, blue, or RGB), consumer-grade devices are often too limited. We present a methodology to increase the number of spectral bands in SFDI devices that use RGB components without hardware modification.

**Approach:** We developed a compact low-cost RGB spectral imager using a color CMOS camera and LED-based mini projector. The components’ spectral properties were characterized and additional cross-channel bands were calculated. An alternative characterization procedure was also developed that makes use of low-cost equipment, and its results were compared. The device performance was evaluated by measurements on tissue-simulating optical phantoms and *in-vivo* tissue. The measurements were compared with another quantitative spectroscopy method: spatial frequency domain spectroscopy (SFDS).

**Results:** Out of six possible cross-channel bands, two were evaluated to be suitable for our application and were fully characterized (520±20  nm; 556±18  nm). The other four cross-channels presented a too low signal-to-noise ratio for this implementation. In estimating the optical properties of optical phantoms, the SFDI data have a strong linear correlation with the SFDS data ($R^{2}=0.987$, RMSE=0.006 for $\mu_{a}$, $R^{2}=0.994$, RMSE=0.078 for $\mu_{s}^{\prime }$).

**Conclusions:** We extracted two additional spectral bands from a commercial RGB system at no cost. There was good agreement between our device and the research-grade SFDS system. The alternative characterization procedure we have presented allowed us to measure the spectral features of the system with an accuracy comparable to standard laboratory equipment.

## 1 Introduction

Biomedical imaging using light can be a powerful, noninvasive, and inexpensive tool to detect biological markers associated with metabolic functions or diseases.^1^ Spectral imaging can be used to gain structural information about the tissue from its diffuse scattering contrast and chemical information by analyzing its absorbance spectrum. Spatial frequency domain imaging (SFDI) is a technique to perform quantitative spectral measurements over an adjustable field of view (FOV) (in the order of 1 to 10 cm), and it is rapidly gathering attention from the scientific community.^2^^,^^3^ The main advantage of SFDI compared to other diffuse optical techniques is the ability to separate the effects of scattering and absorption based on their spatial frequency-dependent effect on reflectance. It also allows one to quantitatively measure chromophores concentrations in turbid media, such as tissues and tissue-simulating phantoms.^4^ Another advantage of this technique is its depth sensitivity depending on the spatial frequencies employed, which allows one to reconstruct a three-dimensional map of optical properties.^5^ SFDI has been employed in clinical studies for a number of different applications, such as vascular assessment in patients with diabetes,^6^ evaluation of burn wounds severity,^7^^,^^8^ enhancement of cancer detection *in vitro*,^9^^–^^11^ and skin characterization for cosmetic surgery.^12^^–^^14^

In several of these applications, measuring only melanin and oxy-/deoxyhemoglobin levels is not sufficient as it is necessary to consider the presence of other chromophores (e.g., met-hemoglobin in burn wounds and carotenoids), which may confound the interpretation of the spectral data. For this reason typical color cameras, which only provide spectral information from three wide bands in the visible range (i.e., red, green, and blue, commonly known as RGB), are not suitable as they can introduce severe underestimation of other important biomarkers (melanin and hemoglobin species).^14^^,^^15^ A number of solutions to improve the spectral sensitivity of the SFDI system have been proposed. However, these involve building customized light sources^16^^–^^18^ or using multispectral cameras/color filters.^7^^,^^19^^,^^20^ A different kind of approach is provided by a technique called spatial frequency domain spectroscopy (SFDS), where the imaging system is replaced by a spectrometer, providing very high spectral resolution at the cost of a greatly reduced FOV and spatial resolution.^15^^,^^21^^,^^22^

A non-negligible aspect of spectral imagers is the cost, and typical optical components and laboratory instrumentation are quite bulky and expensive (in the order of several thousands of dollars). In order to deploy them in clinical or low-resource settings, it is preferable to employ low-cost, portable components. We present an approach to improve the spectral resolution of a nonmodified commercial RGB projector and an RGB CMOS camera in the development of a low-cost compact SFDI system. The signal in the three main channels in such systems (RGB) is given by the combination of the light spectrum and the characteristic of the three color filters. As the spectra of the LEDs are several tens of nm wide, some of the spectral components from one LED (e.g., green) can be also detected by the other color channels in the camera (e.g., red and blue). By shining one LED at a time, the amount of light for all combinations of LEDs/filters was measured, and the spectral overlap was characterized, obtaining up to six additional spectral bands that we will refer to as cross-channel bands. The performance of the system was compared to a research-grade SFDS system, by measuring the optical properties of Intralipid^®^ phantoms and *in vivo* tissue.

## 2 Materials and Methods

### 2.1 Instrument Design

**Fig. 1:**
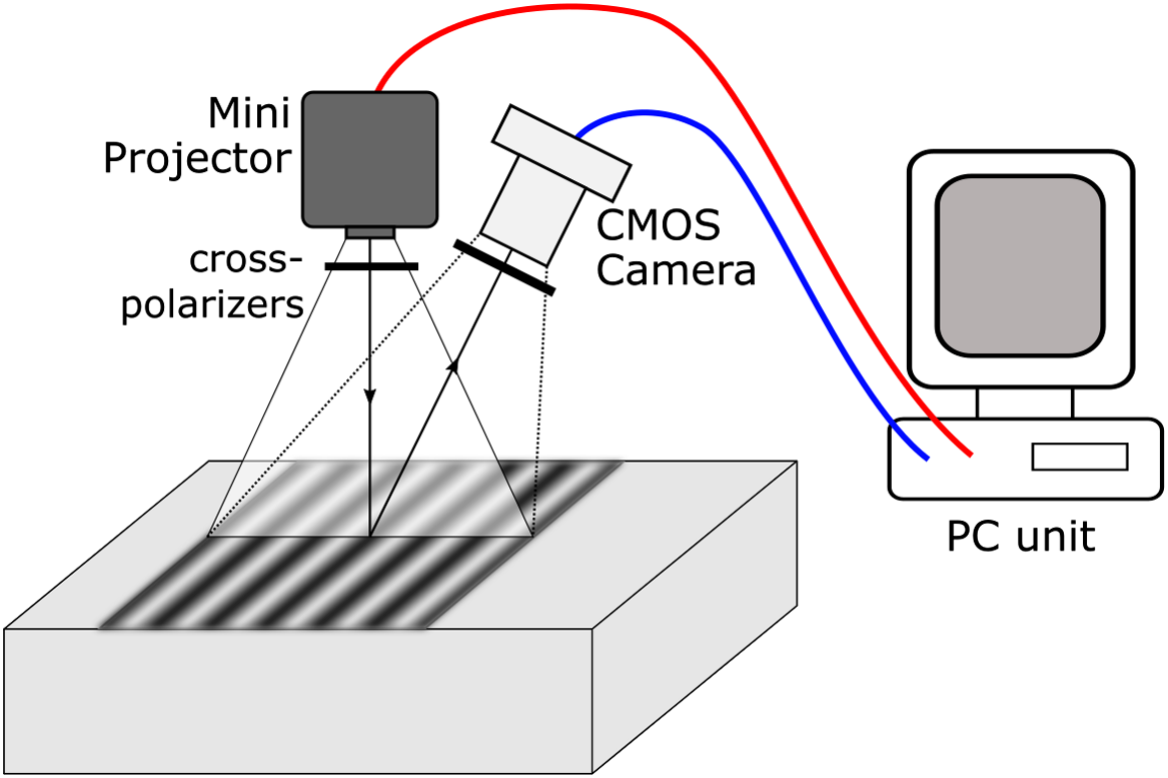
Schematic of the acquisition device. A mini projector is used to shine a sinusoidal pattern on the tissue, then a CMOS digital camera captures the reflected light. Both components are controlled by a computer, and cross-polarizers are placed in front of them to remove specular reflected light.

The developed quantitative imaging device consists primarily of two commercially available components controlled by a computer: a digital LED miniprojector (Innoio, SmartBeam) and a 1.3-MP USB2 color camera (PointGrey, Firefly MV) with a C-mount 12-mm lens (Edmund Optics, UC series). The camera and projector are mounted in a fixed position relative to each other, as shown in Fig. 1, so that they have essentially the same focal plane and FOV. In this setup, we have a working distance of 15 cm, which gives us an FOV of ∼10×5  cm. We are not able to control the projector digital micromirror device directly, so it was employed as a second monitor where the spatially modulated patterns are projected full-screen as images on it. This is a common practice in other low-cost SFDI systems.^22^^,^^23^ The camera was controlled with the software development kit (SDK) provided by the manufacturer (PointGrey, FlyCapture SDK), and the whole acquisition procedure was controlled with Python scripts. Since it is not possible to synchronize the camera with the projector, the exposure time during acquisition should be a multiple of the inverse of the frame-rate (1/60  Hz) in order to avoid flickering in the image intensity.^23^ In order to block specularly reflected reflected light, two cross-polarisers have been applied in front of the camera, and the projector rotated by 90 deg with respect to each other.

### 2.2 Spectral Characterization

The spectral specifications of these devices are not known, and there might also be variability between components in the same series. For this reason, it is necessary to characterize them individually, to know their exact spectral features. The main spectral bands and the cross-channel bands have been characterized using two different approaches, one using laboratory equipment and another using alternative low-cost components. This way it was possible to compare the results obtained with the two methods and determine if the low-cost approach is reliable enough to be used in research.

#### 2.2.1 Laboratory approach

**Fig. 2:**
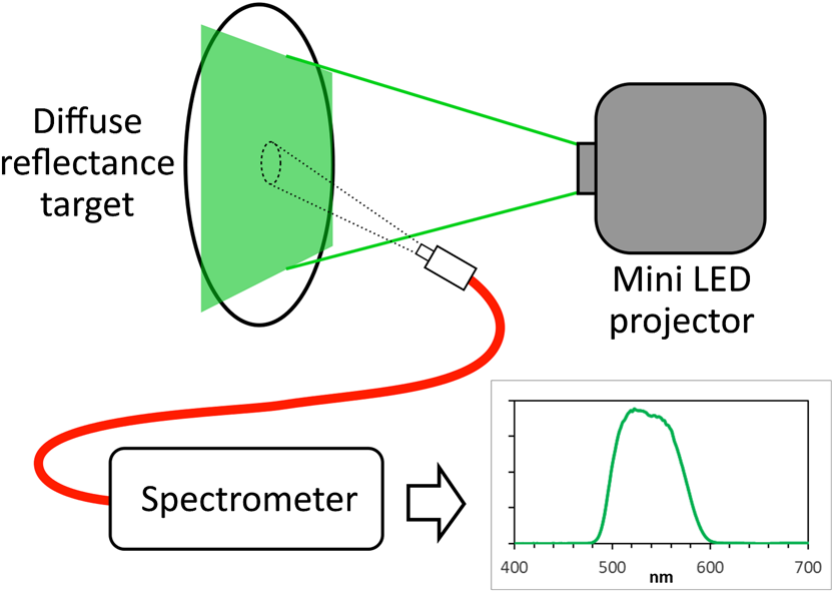
Schematics of the setup used to calibrate the projector: each individual LED shines light on a 99% reflectance reference. The reflected light is captured by an optic fiber and its spectrum is measured with an intensity calibrated spectrometer.

First, an intensity calibration of a spectrometer (Avantes, ULS2048-RS-USB2) was performed, using a calibrated broadband light source (Avantes, Avalight-HAL-CAL-Mini) of which we know the exact intensity over the emission spectrum. To characterize the LEDs, pure red, green, and blue images, respectively, were projected on a 99% reflectance target (Labsphere, calibrated reflectance standard), the reflected light was captured with an optic fiber, and the spectral signature was measured with the intensity calibrated spectrometer (Fig. 2). The spectrum of the three LEDs was normalized to the maximum value of all three bands, in order to preserve the relative intensity ratios.

**Fig. 3:**
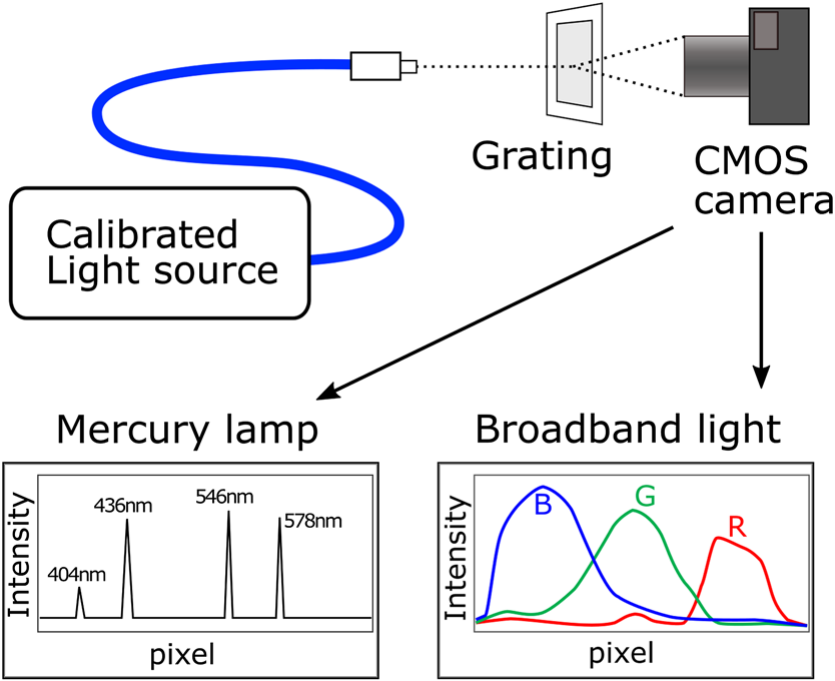
Schematics of the setup used to calibrate the camera: a light source is shone with an optic fiber through a grating, which separates the light in its spectral components along a line. The intensity of the diffracted light is then measured with the camera. A mercury lamp is used for wavelength calibration and a broadband light for intensity calibration.

To measure the characteristic of the camera filters, the following setup was used (Fig. 3): an optic fiber was used to collect light from a light source and concentrate it on a small spot, pointed directly at the camera. A transmission holographic grating (500 grooves/mm) was then placed in front of the camera to separate the wavelengths of the light along a line, and we have acquired images to process. First, a mercury lamp (StellarNet Inc., SL2 calibration lamp) was used to calibrate the wavelengths, as its spectrum has several discrete peaks at known wavelengths that can be used as a reference. To calibrate the intensity, the calibrated broadband white light source was used (Avantes, Avalight-HAL-CAL-Mini). To obtain the characteristic of the red, green, and blue filters on the CMOS sensor of the camera, the intensity profiles of the diffracted light have been analyzed in the red, green, and blue channels of the white light pictures, normalizing their value to the maximum value in all three channels.

Finally, the spectral features of the LEDs and camera that were obtained from this characterization procedure have been combined. The three LED spectra have been multiplied by each of the three color filters function, obtaining this way nine different combinations (the three main color bands and six cross-channel bands).

#### 2.2.2 Low-cost approach

**Fig. 4:**
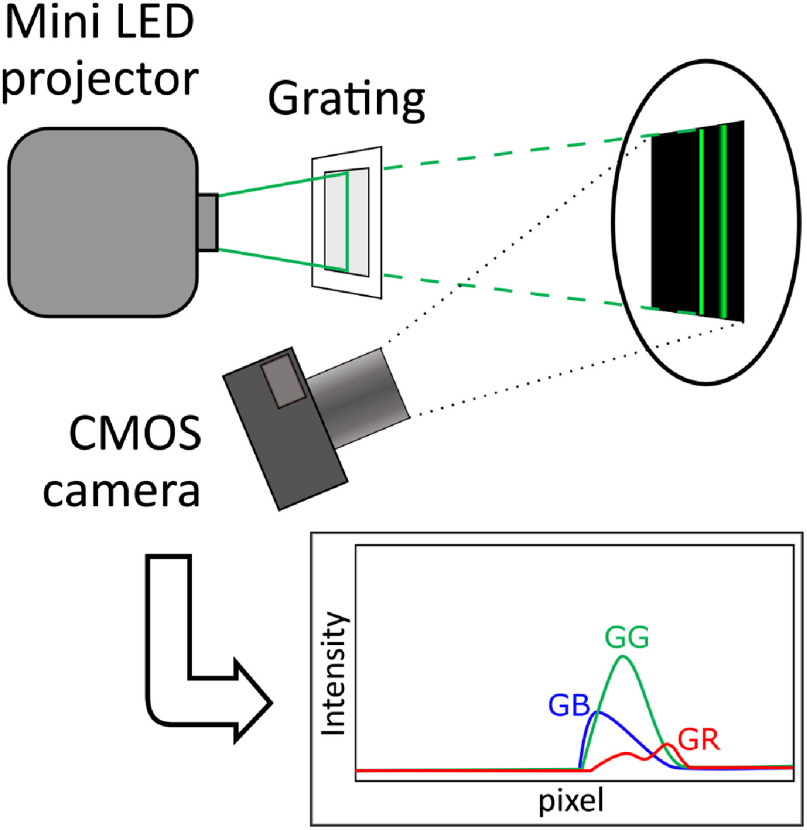
Schematic of the setup used for characterizing the system directly. The projector is used to shine a vertical line of each individual LED color on a 99% reflectance target. The light passes through a grating, which separates its spectral components, and it is captured by the camera. A wavelength calibration is then performed using two laser pointers, in a way similar to Fig. 3.

In the second procedure, the intensity of the cross-channels has been measured directly with the following experimental setup (as presented in Fig. 4). A transmission grating was placed in front of the projector, and a black image with a 1-px vertical line of a pure color (red, green, or blue) was projected on a 99% reflectance target. The grating diffracts the wavelengths horizontally allowing us to interpret the spectral distribution of each LED on the camera directly. The light intensity profiles in each of the color channels of the acquired pictures are a measure of the combined effect of the color filter function and the LED spectrum. A wavelength calibration procedure was performed using two laser pointers of known wavelengths (532 and 650 nm), in a manner similar to the mercury lamp in Sec. 2.2.1, and the signals have been normalized to the maximum value of the each of the three primary LED channels for comparison.

### 2.3 SFDI Acquisition Procedure

To perform SFDI, multiple sinusoidal patterns are projected sequentially on the tissue, and the remitted light (i.e., diffuse reflectance) is measured using the camera. Most commercial projectors, however, use an internal gamma correction to match the nonlinear response of the human eye, and they intentionally alter the brightness levels so that the output is nonlinear. Since pure sinusoidal patterns would be distorted by this gamma correction, it has been characterized and compensated, using the procedure described in previous papers.^22^^,^^23^ For the following data processing steps, it is necessary to measure the amplitude of the AC component of the modulated reflectance. To do so, we employ a multiphase demodulation technique in the spatial domain.^4^ This technique requires the acquisition of three sinusoidal signals with a spatial phase shift of 0 deg, 120 deg, and 240 deg. A simple GUI was developed to control the camera parameters (such as the exposure time), the number of spatial frequencies ($f_{x}$) to generate (at least two) and to start the acquisition in either single acquisition mode or continuous acquisition mode. In this study, patterns at four spatial frequencies (0, 0.0333, 0.05, $0.1\;{\mathrm{mm}}^{-1}$) have been employed. For each $f_{x}$, images were projected at each RGB color and three phases, sequentially, for a total of nine images per $f_{x}$. Then, the individual color channels from the camera needed for our application (3 RGB channels and 2 cross-channels) were extracted and saved in grayscale in uncompressed 8-bit.bmp format. The total acquisition time for an entire sequence was between 10 and 20 seconds, depending on the camera exposure time. The system has not been optimized for speed, however.

### 2.4 Data Processing

The data were processed using the methods described in Ref. 4. The first step to perform is to demodulate the images to obtain the AC amplitude of the signal. Equation (1) has been applied pixel by pixel, where $I_{1}$, $I_{2}$, and $I_{3}$ are the images at a specific $f_{x}$ and spectral band, to obtain the AC amplitude ($M_{\mathrm{AC}}$):

$$M_{\mathrm{AC}}=\frac{\sqrt{2}}{3}\sqrt{{\left(I_{1}-I_{2}\right)}^{2}+{\left(I_{2}-I_{3}\right)}^{2}+{\left(I_{3}-I_{1}\right)}^{2}}.$$ (1)

The second step is the calibration. An SFDI dataset has been acquired under the same experimental conditions on a reference calibration phantom with known optical properties. The aim is to decouple the diffuse reflectance ($R_{d}$) of the tissue from that of the light source intensity ($I_{0}$) and the transfer function of the instrument (${\mathrm{MTF}}_{\text{system}}$). To obtain the diffuse reflectance of the tissue, Eq. (2) was applied at each λ and $f_{x}$:

$$R_{d,\text{tissue}}=\frac{M_{\mathrm{AC}}}{M_{\mathrm{AC},\mathrm{ref}}}\cdot R_{d,\mathrm{ref}},$$ (2)

where $M_{\mathrm{AC}}$ and $M_{\mathrm{AC},\mathrm{ref}}$ are the demodulated amplitudes from the tissue and our reference phantom and $R_{d,\mathrm{ref}}$ is the theoretical reflectance value of the phantom, calculated from the reference’s known optical properties using a forward model of light transport. The model used was a white Monte Carlo model.^24^

The third step is the fitting for optical properties. An inverse solver approach was used to minimize errors between the measured $R_{d,\text{tissue}}f(x)$ and forward Monte Carlo simulations of $R_{d}(f_{x})$ for specific pairs of $\mu_{a}$ and $\mu_{s}^{\prime }$. In this investigation, an iterative Nelder–Mead optimization algorithm (from the scipy.optimize library) then searches in the parameters’ space for a global minimum that minimizes the difference and gives as a result a pair of ($\mu_{a}$, $\mu_{s}^{\prime }$) values.

### 2.5 Optical Phantom Validation Measurements

The device performance was first tested on tissue-simulating liquid phantoms and compared to a validated SFDS system,^21^^,^^25^^,^^26^ used as a reference. Sixteen phantoms were manufactured by combining four different concentrations of Intralipid^®^, which was used as a scattering agent,^27^^,^^28^ and four concentrations of India ink, which was used as an absorber.^27^^,^^29^ The Intralipid^®^ concentrations were (0.5%, 1%, 1.5%, 2%), giving measured values of $\mu_{s}^{\prime }$ comprised in the range 0.5 to $2.5\;{\mathrm{mm}}^{-1}$ @625 nm. The India ink was first sonicated to ensure an homogeneous distribution of the pigment and was added in concentrations of (13, 27, 40, and 53  mg/L), giving measured absorption values in the range 0.025 to $0.25\;{\mathrm{mm}}^{-1}$ @625 nm. A dataset of 60 images (three phases, four spatial frequencies, and five spectral bands) was acquired on all 16 phantoms. Analogously, a dataset of 15 signals (three phases, five spatial frequencies, spanning the spectrum range 400 to 700 nm) was acquired in parallel with the SFDS system. The spatial frequencies used in the SFDS system are (0, 0.05, 0.1, 0.15, 0.2)${\mathrm{mm}}^{-1}$. Under the same experimental conditions, two datasets were also acquired for calibration with the SFDI and SFDS systems on a reference silicon phantom with known optical properties. The exposure time was adjusted for each measurement so that the measured signal would use the entire dynamic range of the camera while not causing the sensor to saturate.

### 2.6 In Vivo Measurements

*In vivo* measurements were performed on healthy skin of a volunteer with both the SFDI and SFDS system. Written informed consent was obtained from the subject. The arm was laid on an armrest, and a dataset was acquired first with the SFDI system and afterward with the SFDS system on the forearm of the subject and on a calibration silicon phantom, like in sec. 2.5. The measurements were performed with the approval of Linköping ethics committee (Dnr 2018/282-31).

## 3 Results

### 3.1 Characterization of Spectral Bands

**Fig. 5:**
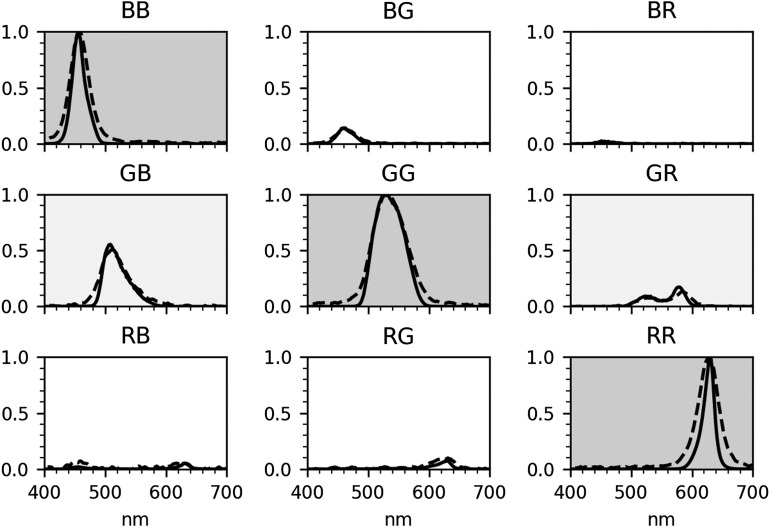
Plot of the nine combinations of the LED and camera spectral features (three main bands on the diagonal and six cross-channel bands). The solid lines are the signals calculated with the laboratory approach, and the dashed line is calculated with the low-cost approach. The rows represent the LEDs color (from the top: blue, green, and red) while the columns represent the camera filters (from the left: blue, green, and red).

The nine spectral bands characterized in Sec. 2.2 are presented in Fig. 5. The naming convention for the spectral bands uses two letters: the first one is referring to the spectral band emitted by the projector (red, green, or blue) and the second one to the filter band of the camera (likewise, red, green, or blue). The solid line is the data derived from the laboratory approach (Sec. 2.2.1), whereas the dashed line is derived from the low-cost approach (Sec. 2.2.2). Based on the data in Fig. 5, two of the cross-channel bands (GB and GR) were determined to be the strongest candidates for our application in term of signal-to-noise ratio (SNR). The BG band also had an intensity level comparable to the GR band, but the absorption of blue light in tissue is very strong, which further degrades the actual SNR of the band. For this reason, we decided not to include the BG band in this study. The signal from the remaining three bands (BR, RB, and RG) was too weak to be useful in this implementation. The central wavelength ($\lambda_{0}$) and full width half maximum (FWHM) of the nine bands were calculated and the main five are reported in Table 1. Since the spectral bands might have a complex shape, these values were calculated by doing a weighted average of the signals, rather than by fitting to a Gaussian function.

**Table 1:** List of the five main spectral combinations in this study, obtained with the two approaches. The values are expressed as ($\lambda_{0}\pm \frac{1}{2}\mathrm{FWHM}$). The naming convention is a letter that indicates the color of the LED followed by a letter that indicates the color of the filter.

Band	Laboratory approach	Low-cost approach
BB (nm)	458±12	465±18
GB (nm)	520±20	519±25
GG (nm)	536±31	535±33
GR (nm)	556±18	561±29
RR (nm)	626±10	617±20

### 3.2 Optical Phantom Validation Measurements

**Fig. 6:**
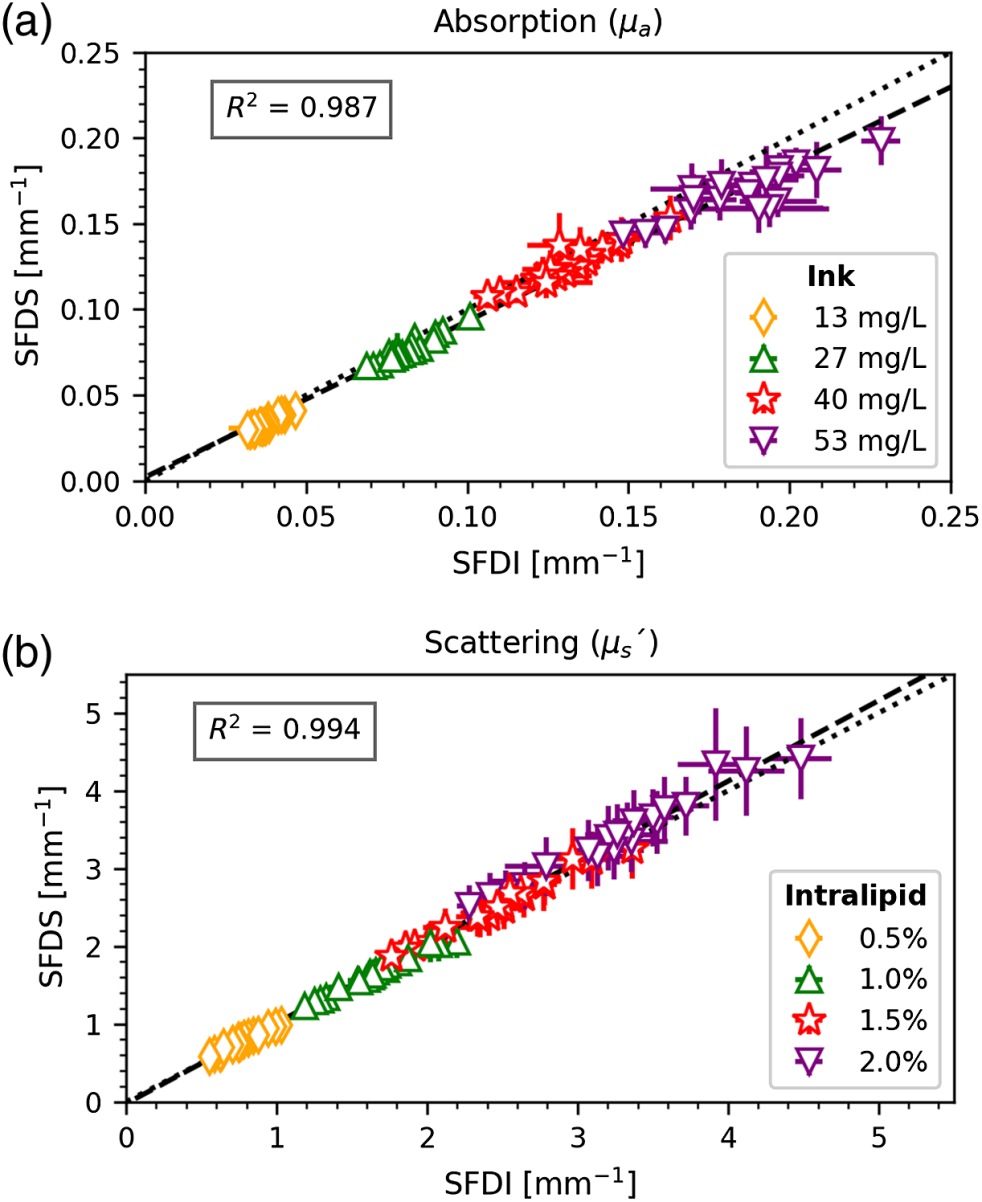
Comparison of the measured values of (a) $\mu_{a}$ and (b) $\mu_{s}^{\prime }$ between the SFDI and the SFDS systems in the five bands, measured on optical phantoms with four levels of absorption and scattering. The dashed lines are the linear regression lines ($R^{2}$ in figure), the dotted lines are the unity lines.

The data were processed with the procedure describe in Sec. 2.4, obtaining a map of $\mu_{a}$, $\mu_{s}^{\prime }$ at five wavelengths with the SFDI system and the whole optical spectrum in a single central location with the SFDS system. Since the phantoms are homogeneous, an average of the optical properties over the whole FOV of the SFDI system was calculated in order to compare it with the SFDS data. Likewise, since the SFDS data are a continuous spectrum, in order to compare it with the SFDI data, spectral properties at the five wavelengths were emulated from the SFDS data using a method described in a previous publication.^14^ The comparison can be seen in Fig. 6, where a linear regression between the values of $\mu_{a}$, $\mu_{s}^{\prime }$ in SFDI and SFDS data is calculated. The color-coded clusters on the graph represent the four different levels of absorption (upper image) or scattering (lower image), which were measured on the phantoms. Each cluster consists of 20 data points, corresponding to the four phantoms that have the same optical property (e.g., same $\mu_{a}$, but different $\mu_{s}^{\prime }$), measured at the five spectral bands. The $R^{2}$ and root mean square error (RMSE) values are reported ($R^{2}=0.987$, RMSE=0.006 for $\mu_{a}$, $R^{2}=0.994$, RMSE=0.078 for $\mu_{s}^{\prime }$).

### 3.3 In Vivo Measurements

**Fig. 7:**
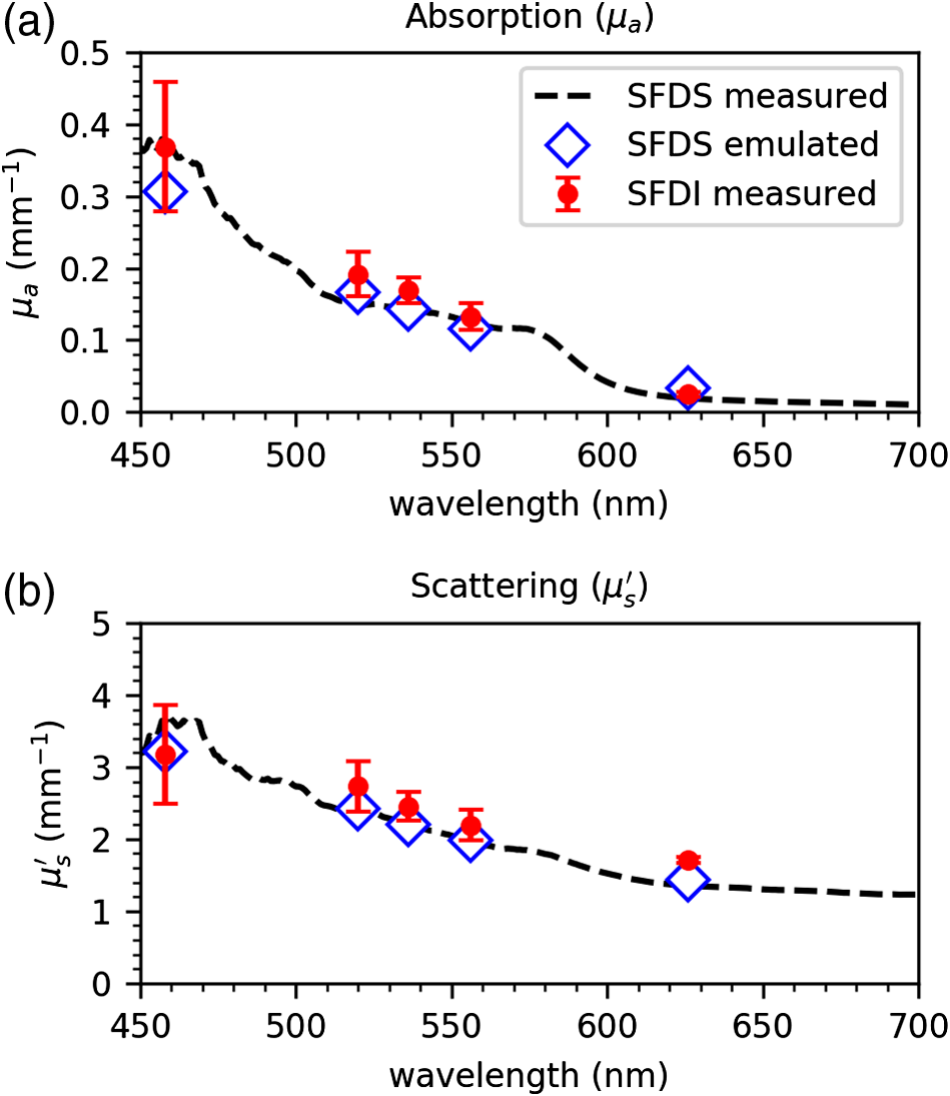
Comparison of the (a) $\mu_{a}$ and (b) $\mu_{s}^{\prime }$ values measured *in vivo* on human forearm skin using SFDI (red circles) and SFDS (dashed line). Five data points are then emulated from the SFDS data, by doing a weighted average over the five bands (blue diamonds).

The data from the *in vivo* measurements were processed in a similar way at the same three spatial frequencies 0, 0.05, $0.1\;{\mathrm{mm}}^{-1}$ both for SFDI and SFDS. This choice of a subset of the acquired $f_{x}$ was made considering the layered nature of skin. This way we are sampling the same volume of tissue at each $f_{x}$ with both systems. The results are shown in Fig. 7. The SFDI data points (red circles) are averaged spatially over the entire image. The SFDS spectrum (dashed line) is averaged spectrally for comparison, by doing a weighted average over the five bands of interest. We obtain five data points (blue diamonds), which emulate what the SFDS system would detect over a broad band, as described in a previous publication.^14^

## 4 Discussion

We have presented a method to extract additional spectral bands from commercial devices, by taking advantage of the spectral cross-talk between the RGB channels of the light source and the detector. This will introduce a significant improvement in the performance of these low-cost devices as spectral imagers, without the need to modify them or employ additional components. In the presented device, the additional cross-channels are situated in the spectral range 500 to 600 nm, which is also where key absorption features of oxy- and deoxyhemoglobin are. This suggests that an improvement in the measurement of blood oxygen saturation and differentiation from other tissue chromophores in the visible regime can be expected.^14^^,^^30^ We also have presented two different methods to characterize the SFDI system: one relying on standard laboratory equipment (price range on the order of $10,000) and a second one, which makes use of common, nonexpensive components (on the order of $10 or less). We can see in Fig. 5 that the spectral resolution of the second method is slightly worse (we have measured a difference in FWHM up to 20 nm). However, the data show good correspondence when compared to the first method. This could represent a reasonable alternative approach for system characterization in low-resource environments, where laboratory equipment is not readily available.

The data in Figs. 6 and 7 show that there is a good correspondence between the values measured with the SFDI and the SFDS system. The $\mu_{a}$ values are clustered in four distinct regions, corresponding to the level of absorption, with the data becoming more noisy at higher absorption. The $\mu_{s}^{\prime }$ values are also clustered in four groups corresponding to the levels of scattering, even though they are more spread out and overlapping because the scattering changes with the wavelength, following a power law of the kind: $\mu_{s}^{\prime }(\lambda )=a\lambda^{-b}$. In the physiological range of optical properties values that we have considered ($\mu_{a}$: 0.05 to $0.25\;{\mathrm{mm}}^{-1}$, $\mu_{s}^{\prime }$: 0.5 to $5\;{\mathrm{mm}}^{-1}$), the measurements are accurate enough to perform SFDI and measure absorption and scattering on skin *in vivo*, as shown in Fig. 7. Regarding the *in vivo* measurements, some considerations should be made on the appropriate choice of spatial frequencies. When using a higher $f_{x}$, light penetrates less in the tissue and a more superficial region is sampled. This has little effect when measuring a target with homogeneous optical properties, as was shown in Fig. 6 where we perform measurements on liquid phantoms using two different sets of $f_{x}$ and obtain highly agreeing values. However, the choice of spatial frequencies is important for the *in vivo* data, due to the layered nature of the tissue. Therefore, processing these data with the same set of spatial frequencies will minimize differences between the two systems due to the effect of spatial frequencies on sampling depth in the layered tissue.

Another issue that is relevant for clinical use is the correction for the curvature of the tissue, which introduces an error in the estimation of optical properties. In context of this study, we have measured a flat region of the arm *in vivo* to minimize this problem. However, methods for surface profile correction have been developed for SFDI techniques and can be easily implemented in an imaging system, such as what is presented here.^31^^–^^33^

The device is light and small and currently needs to be plugged in a computer or laptop, but further improvements are possible. It can be made self-contained by setting up an appropriate developing environment on a small board (e.g., Raspberry Pi) since all code is written in Python and is cross-platform. We have arbitrarily selected this camera mainly because of the ease of control via software with its proprietary SDK, but even smaller and less expensive options are available on the market. Integration of the system with a smartphone (using the phone camera) is also a possibility. Likewise, we have chosen the projector among a few other alternatives because it was the one with the least amount of internal color correction and had a green LED with a wide spectrum, which was preferable for extracting the cross-channel bands. Its price range is around $400, but cheaper and higher resolution portable projectors can be employed.

## 5 Conclusion

We have presented the design and characterization process of a low-cost SFDI system that uses nonmodified commercial components. We have also presented an alternative characterization procedure that makes use of low cost components (transmission grating, laser pointers), as opposed to research-grade instrumentation (spectrometer, calibrated light source). In addition to this, we have developed an approach to extract additional wavelengths from a common RGB system without the need for customized light sources or filters, improving this way its spectral resolution at no cost. These new methodologies will contribute to the development of SFDI in low-resource settings. This will be beneficial both from a clinical perspective, giving clinicians an inexpensive and reliable tool for skin diagnosis, and from a research perspective, promoting the use of the SFDI technique and increasing the possibilities for cooperation and innovation in the field of spectral imaging.
